# Effects of simulated space environmental conditions on cleanroom microbes

**DOI:** 10.3389/fmicb.2025.1600106

**Published:** 2025-08-19

**Authors:** Chelsi D. Cassilly, Atul M. Chander, Jason A. Vaughn, Kevin J. Kunstman, Stefan J. Green, Kasthuri Venkateswaran, Peter F. Bertone, Curtis W. Bahr, Samantha A. Marcella, Heather C. Morris

**Affiliations:** ^1^NASA Marshall Space Flight Center, Materials and Processes Laboratory, Huntsville, AL, United States; ^2^Department of Biology, University of Mississippi, Oxford, MS, United States; ^3^Genomics and Microbiome Core Facility, Rush University, Chicago, IL, United States; ^4^Biotechnology and Planetary Protection Group, Jet Propulsion Laboratory, Pasadena, CA, United States; ^5^Amentum Space Exploration Division, Huntsville, AL, United States; ^6^GeoControl Systems, Huntsville, AL, United States

**Keywords:** whole genome sequencing, radiation, planetary protection, non-spore-forming bacteria, Pan-Resistomics

## Abstract

**Introduction:**

Microorganisms can have major impacts on the success of NASA’s missions, including the integrity of materials, the protection of extraterrestrial environments, the reliability of scientific results, and maintenance of crew health. Robust cleaning and sterilization protocols for spacecraft and associated environments are currently in place in NASA facilities, but microbial contamination should be further controlled and its impact on NASA’s missions and science must be minimized. To address this, air and surfaces across cleanrooms and uncontrolled spaces at the Marshall Space Flight Center were sampled and microbial burden and diversity were analyzed.

**Methods:**

A library of 82 microbial strains was isolated, curated, characterized, and a subset (*n* = 24) was subjected to simulated space environmental stressors, including desiccation, vacuum, proton radiation, and ultraviolet radiation. Out of these, four non-spore-former species (*Arthrobacter koreensis* PPS68, *Paenarthrobacter* sp. PPS72, *Mycetocola* sp. PPS117, and *Erwinia* sp. PPS120) exhibiting the highest resistance to tested stressors were selected for whole genome sequencing and comparative genomic, pan-resistomics and functional analyses.

**Results:**

The analysis revealed genomic features among these four species, encompassing genes critical for amino acid biosynthesis, carbohydrate metabolism, and stress response mechanisms. *Erwinia* sp. PPS120 had genomic features indicative of metabolic flexibility and stress response capabilities, particularly under oxidative stress conditions. Notably, strain *A. koreensis* PPS68 had unique genomic features predictive of resilience to desiccation and ionizing radiation, supported by genes for oxidative stress resistance, membrane stability, and nutrient acquisition. *A. koreensis* contains several genes which are also reported in established radioresistant strains, for predicted functions related to DNA-repair, osmoprotection, and efflux.

**Discussion:**

NASA cleanrooms harbor hardy non-spore-forming bacteria capable of surviving vacuum, ionizing radiation, and UV. Their adaptations to space stressors suggest limitations of today’s spore-centric bioburden assays to explore expanded planetary-protection standards. The modular exposure assay and reference genomes are important resources for microbial risk assessment, decontamination design, and safeguarding both robotic missions and closed human habitats in space and earth where microbial presence and colonization could compromise life-support systems and crew health.

## 1 Introduction

Microbiology within National Aeronautics and Space Administration (NASA) typically falls into a handful of disciplines that include but are not limited to Space Biology, Environmental Control and Life Support Systems (ECLSS), Astrobiology, and Planetary Protection (PP). As NASA continues the exploration of our solar system, both robotically and in complex crewed missions, understanding the microbes that pose the greatest risk to mission success is of utmost importance. In robotic missions to certain target bodies of interest, like Mars, it is critical to prevent microbial contamination that could adversely impact scientific objectives (Olsson-Francis et al., 2023). Specifically, NASA PP policy requires stringent cleanliness protocols to be in place to remove most microbes from spacecraft surfaces and volumes, with the level of expected cleanliness increasing for missions landing on sensitive solar system bodies. However, the methods used to quantify cleanliness are largely based on the cultivation of bacterial spores [i.e., NASA Standard Assay (NSA, NASA-HDBK-6022, 2010) and provide information neither about the risk of contaminating target bodies (e.g., are putative extremophiles present?) nor appropriate mitigation strategies (e.g., is heat required, or will an ethanol wipe suffice?)].

Each cleanroom and spacecraft assembly facility is unique, and it is therefore important to characterize the microbial populations present in such environments to understand potential PP risks. Several studies have identified persistent microbial species in NASA cleanrooms and spacecraft assembly facilities, with some non-spore-forming species exhibiting resistance to radiation, desiccation, and cleaning protocols. For example, *Tersicoccus phoenicis*, was first identified in NASA’s Phoenix Mars Lander cleanroom in 2007 (Vaishampayan et al., 2013). This non-spore-forming bacteria thrives in nutrient-poor conditions and was found in two separate cleanrooms 2,500 miles apart, suggesting that cleanroom-adapted microbes can spread within these specialized environments. Similarly, *Deinococcus phoenicis*, a radiation-resistant bacterium isolated from the Phoenix Lander assembly facility at NASA’s Kennedy Space Center, has been shown to survive extreme gamma radiation (D10 > 8 kGy) and UV exposure [D10 > 1,000 Jm^−2^], demonstrating that non-spore-forming extremophiles may pose PP related risks (Vaishampayan et al., 2014). This is particularly concerning because PP protocols primarily target spore-forming bacteria, potentially overlooking hardy non-spore-formers like *Deinococcus* (Mattimore and Battista, 1996). Additional bacterial species have been repeatedly isolated in spacecraft assembly facilities, reinforcing the importance of understanding microbial persistence in these environment. *Acinetobacter radioresistens*, was cultured from the Mars Odyssey orbiter during assembly-facility (La Duc et al., 2007), while *Brevundimonas diminuta*, a Gram-negative bacterium emerged as a predominant species following intensified cleaning regimes during the Phoenix Lander assembly (Ghosh et al., 2010). The resilience of *B. diminuta* in nutrient-poor, disinfected environments suggests it may persist despite rigorous sterilization measures, has made it a model organism for PP studies (Kimura et al., 2023). Furthermore, spore-forming bacteria, particularly *Bacillus* and related genera (*Paenibacillus*, *Geobacillus*, *Oceanobacillus*), remain classic contaminants in cleanrooms, with numerous strains being recovered from both NASA and ESA facilities (La Duc et al., 2007; Moissl-Eichinger et al., 2012).

For crewed missions beyond low Earth orbit, microbial adaptations to high-radiation environments could impact microbial survival or physiology, which may impact crew health or other scientific objectives. It is important to investigate microbial survival and adaptation under space-like stressors, including desiccation, vacuum, and proton radiation. Previous studies have evaluated the effects of space stressors on human-associated microorganisms (Tarpley et al., 1953; Cao et al., 2015; Bruckbauer et al., 2020; Cortesão et al., 2020), liquid cultures (Beblo-Vranesevic et al., 2017; Verbeelen et al., 2024), and spore-forming bacteria (Schuerger et al., 2003; Khodadad et al., 2017; Cortesão et al., 2019; Deng et al., 2023). However, little is known about the effects of deep-space-like stressors on dried, non-spore-forming cleanroom isolates (Osman et al., 2007; Dance, 2025).

The scope of this project was to determine the effects of space-like stressors, including desiccation, vacuum, and proton radiation, on dried microbes isolated from various locations at the Marshall Space Flight Center (MSFC) with varying degrees of cleanliness. Given that each cleanroom is unique, investigating the microbial composition at MSFC is critical for assessing potential contamination risks. We performed multiple studies at several proton fluence levels and used the resultant data to narrow our test group for subsequent tests. Selected PP-relevant candidate strains were subjected to whole genome sequencing (WGS) and comparative genomic and functional analyses.

## 2 Materials and methods

### 2.1 Microbial collection and isolation

Four cleanrooms at MSFC were selected for sampling together with two uncontrolled lab spaces. One cleanroom is not maintained and certified as a cleanroom but still has controlled access. In all cases, air samples were collected into 10 mL sterile water using a Coriolis *μ* air sampler (Bertin, Montigny-le-Bretonneux, France). Subsequently, field control samples were collected by waving a swab through the air for approximately 5 s. Surface samples of small area (5″ by 3″) using either dry or sterile water-wetted swabs (806WC, Puritan) or large area (27″ by 27″) were collected using 9″ by 9″ sterile water-wetted wipes (TX3211; TexWipe, Kernersville, NC).

Air samples (300 L/min) were collected for 5 min into 10–15 mL sterile water. After completion, collection cones were capped and stored up to 7 days at 4°C until processed. Swabs were collected and then stored dry in sterile tubes at 4°C for up to 7 days until processed. Wipes were dampened in 10 mL sterile water within a sterile petri dish and following collection, stored in sterile glass jars at 4°C up to 27 days until processed.

Tryptic Soy Agar (TSA) (BD Biosciences, Franklin Lakes, NJ) and Tryptic Soy Broth (TSB) (BD Biosciences, Franklin Lakes, NJ) were used as media for cultivation of the bacteria. All swabs were streaked directly onto the surface of a TSA plate and then incubated at 32°C for at least 2 days. Wipes were submerged in 35 mL of sterile water and then sonicated. A volume of 250 μL was then pipetted onto TSA, spread using a sterile spreader and incubated at 32°C for at least 2 days. Last, 100 μL of liquid collected via Coriolis air sampler was spread on TSA using sterile spreaders and incubated at 32°C for at least 2 days. In most cases, if overgrowth was observed, or too numerous colonies to count (TNTC), dilutions were made of the liquid into sterile water and replated until countable colonies were observed.

Strains of *Bacillus atrophaeus* ATCC 9372 and *Deinococcus radiodurans* ATCC 13939 purchased from American Type Culture Collection (ATCC) were used as positive controls. Sterile water (*n* = 3) and DNA purification kit blanks (*n* = 3) were employed as negative controls.

### 2.2 Microbial library curation

For all plates, representative colonies for all morphologies observed were selected and given a unique identifier. Selected colonies were all streaked to confirm purity on TSA and incubated at either 25°C or 32°C depending on whether the colony was classified as fungi or bacteria, respectively, until sufficient growth and isolated colonies were observed for subsequent experiments. If multiple colony morphologies were detected, single colonies were selected and streaked again. If molds were observed and single colonies could not be obtained, the isolates were sub-cultured until only a single morphology was detected.

For the physical curation, when possible, strains were subcultured from one colony using a sterile loop into 1.5–3 mL TSB and grown stationary and incubated at either 25°C or 32°C. When turbid growth was observed, the strains were frozen with a 1:1 volume of 25% sterile glycerol in cryotubes and stored at −80°C. If an organism was unable to grow in liquid culture, colonies from the TSA plates were scraped with a sterile loop into a 1:1 mixture of TSB:25% glycerol and frozen. All frozen cultures were then re-streaked on TSA with a sterile loop and grown at 25°C or 32°C until growth was seen. If no growth, a mixed culture, or a culture with unmatched description was observed, the culture was discarded, and renewed attempts were made to obtain a pure culture from the original plates. It was only after a stock culture was frozen and the growth was verified that the original plates were discarded. All strains were classified as either suspected fungi or suspected bacteria based on colony morphology.

### 2.3 DNA extraction

DNA extraction was conducted using a Quick-DNA Fungal/Bacterial Miniprep Kit (Zymo Research, D6005, Irvine, CA). To collect pellets, pure cultures were scraped from TSA plates using sterile loops and deposited into a weighed microcentrifuge tube. The tubes were centrifuged at maximum speed for 1 min in an accuSpin Micro 17 Microcentrifuge (13-100-675; Thermo Fisher Scientific, Waltham, MA). The microcentrifuge tubes were weighed, and pellet weights were recorded. Pellet wet weights of 50–100 mg were targeted and multiple plates were used if necessary to get sufficient biomass. Pellets were stored in a −20°C freezer until ready for processing. For DNA extraction, pellets were thawed in a biosafety level 2 (BSL2) hood and the manufacturer’s instructions were followed for isolating genomic DNA. DNA concentration was measured using a Thermo Scientific Nanodrop Lite (Waltham, MA).

### 2.4 Sanger sequencing and DNA analysis

Microbial taxonomic characterization was performed using capillary electrophoresis (i.e., Sanger) sequencing of small subunit (SSU; 16S or 18S) ribosomal RNA (rRNA) gene amplicons generated from PCR amplification of genomic DNA using bacterial or fungal primer sets. Off-the-shelf primers for bacterial 16S rRNA gene amplification (1,500 bp amplicon) were purchased from Integrated DNA Technologies (IDT). Fungal primers ITS5F and ITS4R, custom synthesized by IDT (Coralville, IA), were used to amplify internal transcribed spacer (ITS) regions (500 bp amplicon) of suspected fungal isolates (Schoch et al., 2012).

PCR was conducted using an Applied Biosystems SimpliAmp Thermalcycler (A41192; Thermo Fisher Scientific, Waltham, MA). Detailed information can be found in the Supplementary methods.

Amplicons were sequenced by Azenta Life Sciences-Genewiz for Sanger sequencing. FASTQ sequences were downloaded, individually assessed and trimmed of unresolved bases (on average the first 20 to 40 bases and the last 20 to 150 bases depending on the length and quality of the sequence), and annotated using the rRNA/ITS database using the Targeted Loci Project Information from either the 16S ribosomal RNA sequence (Bacteria and Archaea) or ITS from Fungi and type reference material in the National Center for Biotechnology Information (NCBI) Basic Local Alignment Search Tool [BLAST; e.g., (Altschul et al., 1990)]. Genus-level annotation was only assigned when the closest taxonomically annotated reference sequence was at least 97% similar, and species-level annotation was only assigned when reference sequences were >99% similar, and there was a 0.2% difference between the top IDs (Supplementary Table 1).

### 2.5 Microbial culturing for exposure studies

Media used for culturing strains was TSA, Nutrient Agar with 1% glucose (BD Biosciences, Franklin Lakes, NJ), Lennox Broth (LB) agar, and TSB. Frozen stock cultures were scraped with a sterile loop and streaked across the surface of a TSA plate, a LB agar plate, or a nutrient agar plate, and then incubated at 32°C for at least 2 days. Once adequate growth was observed, cells from a single colony were collected with a sterile loop and inoculated into 3 mL of TSB. Cultures were grown either stationary or shaking at 250 rpm at 32°C for at least 2 days or until adequate turbidity was observed. Optical density was measured at 600 nm wavelength using a Genesys 50 UV/VIS spectrophotometer (Thermo Fisher Scientific, Waltham, MA) immediately following vigorous vortexing. Cultures were centrifuged and resuspended in sterile water to an OD_600_ of 0.1 (or approximately 1 × 10^6^ cells/mL). Positive controls *B. atrophaeus* ATCC 9372 and *D. radiodurans* ATCC 13939 were included as needed.

### 2.6 Microbial application and radiation exposure

Kapton-HN film was cut into 1-in^2^ coupons and sterilized using a 3870ELP Heidolph Tuttnauer benchtop sterilizer. While each run was performed with independent cultures prepared specially for that experiment, the data from each run informed the next, meaning that the runs were all conducted differently and are outlined individually below and as described in Supplementary Table 2. Diluted cultures were mixed with a vortex and the specified volume was applied as a single droplet to the surface of a labeled Kapton coupon. All work was performed in a biosafety cabinet. Samples were allowed to dry for up to 3 days. Samples were then transferred to the radiation test facility for exposure. In all cases, media controls without bacteria were similarly exposed. Ambient coupons devoid of microbes but spotted with sterile water (negative control) were transferred between buildings along with the experimental coupons but were unexposed to vacuum or radiation. For the final run, a second set of controls, called vacuum controls, were also subjected to the ~1E-6 Torr vacuum, like the test specimens.

In a series of experiments, microbial cultures were deposited on sterile Kapton coupons and subjected to proton and ultraviolet (UV) radiation to assess their resilience under extreme conditions. The coupons were prepared in a biosafety cabinet, where microbial samples were dried before exposure. Proton radiation experiments were conducted at the Combined Environmental Effects Facility (CEEF) at MSFC. It is difficult to measure the dose of radiation received by such small targets (microbes), and pathfinder studies demonstrated the usefulness of relying on fluence for dosing (Cengel et al., 2010). Therefore, samples were exposed to 100 keV protons at fluences ranging from 2 × 10^15^ to 4 × 10^15^ p+/cm^2^ under high vacuum (~1E-6 Torr). Each run featured minor procedural variations. In the first run, 250 μL of microbial culture was applied, but all samples being irradiated, leaving no controls. Subsequent runs utilized 200 μL to ensure better drying and included ambient unexposed controls. The second, third, and fourth runs proceeded with increased fluences and adjusted exposure times, ranging from 10.7 to 36.5 h, under vacuum durations of 46 to 145 h (Supplementary Table 2).

Additionally, a UV radiation experiment exposed samples to 254 nm light at an intensity of 80 W/m^2^ at a distance of ~18 cm for 5 or 10 min using a Spectroline XL-1500 UV Crosslinker. These samples were processed on the same day to minimize contamination. Instances of contamination were seen only in the first run, and improved sample handling was employed in subsequent runs, improving outcomes.

### 2.7 Post irradiation processing

Following radiation experiments, samples were transferred to a BSL2 lab for further processing. In all cases, sample processing included the transfer of a single Kapton coupon into a sterile 50 mL conical tube. A volume of 5 mL of sterile water was added, and the sample was vortexed vigorously to release cells from the coupon surface. In the case of the first and second runs, 250 μL of eluate was pipetted on appropriate solid media and spread across the surface using a sterile L-shaped spreader. Plates were incubated at 32°C for 2 days. For the last two runs, spot plating was performed with 100 μL. All negative control samples were treated the same way as the experimental samples. An inoculum control was not included for the first run. For the inoculum growth control in the second run, 100 μL was spread plated to verify growth. For the remaining runs, 100 μL of inoculum were pipetted as spots on the agar surface and incubated at 32°C until sufficient growth was observed. Due to the high number of plates, quantitative data was not obtained, rather an arbitrary qualitative system was employed to describe the growth as growth (+), minor growth (minor), no growth (−), or contamination. Growth is defined as colonies observed in three replicate samples per microbe. Minor growth is defined as at least one colony observed in at least one replicate sample. No growth was defined as the lack of observable growth in any sample. Contamination was indicated if microbial colonies differing in appearance from those of the selected isolates were identified. Control coupons were processed identically. Finally, inocula were plated to ensure that adequate starting culture was present and to compare morphology and determine any contamination.

### 2.8 Bacterial whole genome sequencing (WGS)

WGS of strains PPS68, PPS72, PPS117, and PPS120 were carried out as previously described (Popovich et al., 2021). DNA quantity and quality were measured using fluorimetry (Qubit 4; ThermoFisher) and electrophoresis (TapeStation4150; Agilent). Library preparation was performed using a Nextera XT kit (#FC-131-1024; Illumina Inc. San Diego, CA) according to the manufacturer’s instructions with 1 ng template input and 12 cycles of indexing PCR. An equal-volume pool of all libraries was then prepared, and deep sequencing was performed using an Illumina NovaSeq X instrument targeting 5 million clusters per sample (paired-end, 2 × 150 base reads). Draft genomes were assembled using the software package CLC Genomics Workbench (v22; Qiagen). Library preparation was performed at the Genomics and Microbiome Core Facility (GMCF) at Rush University, IL, USA, and deep sequencing was performed at the DNA Services Facility at the University of Illinois at Urbana-Champaign.

### 2.9 Genome assembly and 16S rRNA gene identification and annotation

Bacterial genomes were *de novo* assembled using the CLC Genomics Workbench software (v24) using shotgun sequence data generated on an Illumina sequencer. Default settings were applied for the assembly process, except for the minimum contig length, which was set to 200 base pairs to ensure the inclusion of smaller but potentially important contigs. Using BLAST analysis (i.e., BLASTn-v2.12.0+) against the NCBI’s 16S ribosomal RNA sequences database, 16S rRNA genes were extracted from assembled genome sequences. In addition, the assembled genomes were annotated by Rapid Annotation using Subsystem Technology (RAST) as described previously (Chander et al., 2017). Data for each annotation was exported from the RAST server and further processed through Pan-ResistomeFinder. The “role” column in the RAST exported annotation data shows gene/protein while other columns assign these genes into larger groups based on their function. There may be multiple entries of a single gene/protein in the column “role” based on their multiple roles. To account for the functional diversity of each gene, uniqueness of genes in this column was ignored.

### 2.10 Strain selection for comparative resistome analysis with radioresistant strains of phylum *Actinomycetota*

Strains with experimentally validated D₁₀ values of 2 kGy or higher (for γ-irradiation or equivalent UV-C fluence) were designated as “resistant,” while those below this threshold served as “sensitive” controls (Table 1). The final panel comprises eleven resistant strains *Deinococcus radiodurans* R1 (Daly, 2009), *Deinococcus radiomollis* (Callegan et al., 2008), *Kocuria rhizophila* PT10 (Guesmi et al., 2021b), *Kocuria rosea* DSM 20447 (Asgarani et al., 2012), *Kineococcus radiotolerans* SRS30216 (Bagwell et al., 2008), *Promicromonospora panici* PT9 (Guesmi et al., 2021a), *Rubrobacter xylanophilus* DSM 9941 (Ferreira et al., 1999), *Rubrobacter radiotolerans* DSM 5868 (Ferreira et al., 1999), *Rubrobacter radiotolerans* RSPS-4 (Ferreira et al., 1999), *Geodermatophilus obscurus* DSM 43160, and *Geodermatophilus dictyosporus* DSM 44208 (Montero-Calasanz et al., 2015). The sensitive group includes *Escherichia coli* O157, *E. coli* K-12 MG1655, and *Arthrobacter* sp. PAMC 25486 (KOPRI Repository, n.d.). These additional set of strains provide a robust two-class framework for downstream comparative analysis of gene presence patterns related to radiation resistance.

**Table 1:** Details about radiation resistant strains used for comparative resistome analysis.

Strain name	Radiation type	Classification	D10 Value	ACCESSION#
*Escherichia coli* O157	–	Sensitive Represntative	–	GCF_000005845.2
*Escherichia coli* str. K-12 substr. MG1655	–	Sensitive Represntative	–	GCF_000008865.2
*Arthrobacter* sp. PAMC 25486	Gamma	Sensitive	370 Gy	GCF_000785535.1
*Deinococcus radiomollis*	Gamma	Resistant	2.2 kGy	GCF_045784305.1
*Deinococcus radiodurans* R1	Gamma	Resistant	6-12 kGy	GCA_000008565.1
*Kocuria rhizophila* PT10	Gamma	Resistant	2.9 kGy	GCF_900576785.1
*Promicromonospora panici* PT9	γ-rays (Co-60)	Resistant	2.6 kGy	GCF_900608595.1
*Kineococcus radiotolerans* SRS30216	γ-rays (Cs-137)	Resistant	2.3 kGy	GCF_000017305.1
*Kocuria rosea* DSM 20447	γ-rays (Co-60)	Resistant	0.13–0.25 Mrad	GCF_006717035.1
*Rubrobacter xylanophilus* DSM 9941	Gamma (Co-60)	Resistant	4.6 kGy	GCF_000014185.1
*Geodermatophilus obscurus* DSM 43160	Gamma (Co-60)	Resistant	9 kGy	GCF_000025345.1
*Geodermatophilus dictyosporus* DSM 44208	Gamma (Co-60)	Resistant	8.5 kGy	GCF_900115505.1
*Rubrobacter radiotolerans* DSM 5868	Gamma (Co-60)	Resistant	7.6 kGy	GCF_900175965.1
*Rubrobacter radiotolerans* RSPS-4	Gamma (Co-60)	Resistant	9.0 kGy	GCF_000661895.1

### 2.11 Taxonomic annotation of the selected strain panel

NCBI taxonomy database confirms that, apart from the genus *Deinococcus* (order *Deinococcales*, phylum *Deinococcota*), all other reference strains in the radiation phenotype panel belong to the phylum *Actinobacteriota*. The resistant group includes *Kocuria rhizophila* PT10 and *Kocuria rosea* DSM 20447 from the order *Micrococcales*, *Kineococcus radiotolerans* SRS30216 from *Kineosporiales*, *Promicromonospora panici* PT-9 from *Propionibacteriales*, *Geodermatophilus obscurus* DSM 43160 and *Geodermatophilus dictyosporus* DSM 44208 from *Geodermatophilales*, and *Rubrobacter radiotolerans* DSM 5868, *Rubrobacter radiotolerans* RSPS-4, and *Rubrobacter xylanophilus* DSM 9941 from *Rubrobacterales*. Among the sensitive strains, *Arthrobacter* sp. PAMC 25486 (order *Micrococcales*) also belongs to *Actinobacteriota*, while the two *Escherichia coli* strains, O157 and K-12 MG1655, are members of the phylum *Proteobacteria* (order *Enterobacterales*). This updated composition emphasizes broad phylogenetic coverage while confirming that the majority of strains in the panel, particularly the resistant ones, are taxonomically affiliated with *Actinobacteriota*.

### 2.12 Automated resistome extraction using Pan-ResistomeFinder

For extraction and comparison of stress and resistance-associated genes from multiple microbial genomes, we used Pan-ResistomeFinder (https://github.com/atulchander/Pan-ResistomeFinder). This tool supports automated processing of genomic annotation files in .tsv, .csv, .xls, or .xlsx formats, each corresponding to a different strain. It ensures the presence of core functional descriptors (Category, Subcategory, Subsystem, and Role) and constructs a unified gene presence–absence matrix by merging input files on these shared descriptors. Genes were marked with a binary indicator (1 for presence) across strains, and missing annotations were imputed as 0. The merged matrix captures the distribution of functional genes across the strains. To focus specifically on stress and resistance-related functions, Pan-ResistomeFinder performs a keyword-guided resistome search. This produces a filtered output containing genes relevant to resilience, which is basis for subsequent resistomics. To characterize strain-specific resistome overlap, we implemented a focused extraction protocol using Pan-ResistomeFinder. First, the resistome output matrix filtered for stress and resistance-related genes was queried to identify total resistome in the dataset. In this total resistome dataset, a subset of data was created showing presence of genes in *A. koreensis* PPS68 and simultaneously found in at least one of the confirmed resistant strains. This subset was saved as the “shared resistome” dataset, representing genes potentially conserved in resistance-competent *Actinobacteria.* From this shared subset, we further excluded any genes that were also present in any known sensitive strains (*E. coli* O157, *E. coli* K-12 MG1655, and *Arthrobacter* sp. PAMC 25486). The resulting exclusive set, comprising genes unique to strain PPS68 and resistant strains only, was saved separately to capture PPS68-associated resistome signatures that may be selectively retained in radiation-adapted taxa.

### 2.13 Genome annotation using average nucleotide identity (ANI) analysis

We used Genome Taxonomy Database (GTDB)-Tk v2.4.0 to compute the Average Nucleotide Identity (ANI) between our query genomes and the reference genomes from the GTDB release r220. This analysis aimed to identify the closest representative genome for each query genome based on ANI. Four query genomes in .fna format were analyzed against the comprehensive GTDB reference dataset. The analysis was conducted using the ani_rep command in GTDB-Tk, with the genome directory, output directory, file extension (.fna). The results included two main output files: gtdbtk.ani_summary.tsv, which provided ANI values for all comparisons, and gtdbtk.ani_closest.tsv, which lists the closest representative genome for each query genome.

Further analysis was performed using the FastANI software with default parameters to calculate the Average Nucleotide Identity (ANI) for the four query genomes: strain PPS68, strain PPS72, strain PPS117, and strain PPS120. Output included ANI values, number of fragments mapped, and total fragments analyzed. FastANI analysis was performed by the Rush Research Bioinformatics Core (RRBC) at Rush University. These genomic comparisons were performed between the assembled draft genomes and reference genomes downloaded from the NCBI database. All available NCBI genomes from genera of interest, including GenBank (GCA) and RefSeq (GCF), were downloaded for comparison. Genus-level taxonomic identification was initially confirmed using 16S ribosomal RNA gene BLAST analysis (Altschul et al., 1997; Jain et al., 2018).

## 3 Results

In total, 82 microbial isolates were recovered from four sampling locations at the MSFC with varying levels of cleanliness, including ISO8 locations (A-C) and uncontrolled locations (D-F). As the aim of this study was to isolate and identify cleanroom extremotolerant with the ability to withstand harsh conditions, but not to evaluate cleanroom cleanliness, samples were collected and processed using multiple approaches that differed from the NSA (NSA, NASA-HDBK-6022, 2010). Furthermore, the collection of isolates was limited only to culturable aerobic microbes given that downstream exposure studies required that the strains be cultured. While this narrowed the diversity of microbes recovered, it was beyond the scope of this work to fully evaluate the composition of cleanroom microbes using cultivation and molecular approaches. Based on plate morphology, three were tentatively identified as fungi and the remaining 79 were presumptively identified as bacteria. Of the 82 isolates, 68 strains yielded PCR amplification with bacterial and fungal domain-level primer sets. The remaining 14 isolates were re-streaked on TSA and DNA was re-extracted, enabling genus-level or higher taxonomic identification for all. In total, 71 isolates provided high-quality sequencing data with annotations to the genus level or beyond (Supplementary Table 1). DNA amplification issues were primarily attributed to the misidentification of yeast/fungal strains as bacteria, as yeast lack the 16S rRNA gene required for 16S PCR amplification. For example, of the 14 isolates re-sequenced, five yeasts were initially misidentified as bacteria, but light microscopy confirmed yeast-like morphologies. Additionally, some isolates exhibited sequencing issues, such as high background noise, indicative of insufficient DNA quality or quantity. This may have been due to non-axenic cultures despite multiple purification attempts.

Several strains (*n* = 24) were selected based on their taxonomic identity, growth characteristics, and a targeted literature review conducted using PubMed. The search included keywords such as “radiation resistance,” “spore-forming,” “extremophile,” and the genus or species names of the isolates. Articles were screened for evidence of physiological traits associated with extremotolerance (e.g., resistance to desiccation, oxidative stress, or nutrient limitation), and strains with such attributes were prioritized for further characterization.

To assess their survival under space environmental conditions, a series of ionizing radiation experiments were conducted on a subset of the strains (*n* = 24). Five radiation exposure tests were performed, with the results summarized in Table 2. For the first run, microbial isolates were exposed to 100 keV protons to a fluence of 2 × 10^15^ p+/cm^2^. Of the 24 strains, 13 isolates demonstrated marked survival to this exposure, while seven showed minor growth. For the second run, 13 strains were tested again along with *D. radiodurans* (ATCC 13939) as a positive control. In this run, four strains survived with one showing minor growth and no contamination observed. For comparison, the *D. radiodurans* positive control exhibited only minor growth. In subsequent tests, *B. atrophaeus* (ATCC 9372) was added as a more resistant positive control. For the third run the radiation dose was increased by 50%, and five isolate strains were exposed in addition to *D. radiodurans* and spores of *B. atrophaeus* as positive controls. Three of five strains survived these exposure conditions, along with two instances of minor growth. In addition, *D. radiodurans* demonstrated minor growth, while *B. atrophaeus* spores survived. Similar results were observed in the fourth exposure experiment. In this fourth test, radiation conditions were identical to those of the third run but included two control sample sets: (1) ambient controls in which all taxa were maintained at normal pressure, and (2) vacuum which were subjected to the same level of vacuum as test specimens (Table 2).

**Table 2:** Identification of strains isolated during this study based on 16S or ITS rRNA gene sequencing and survival against various simulated space environmental conditions.

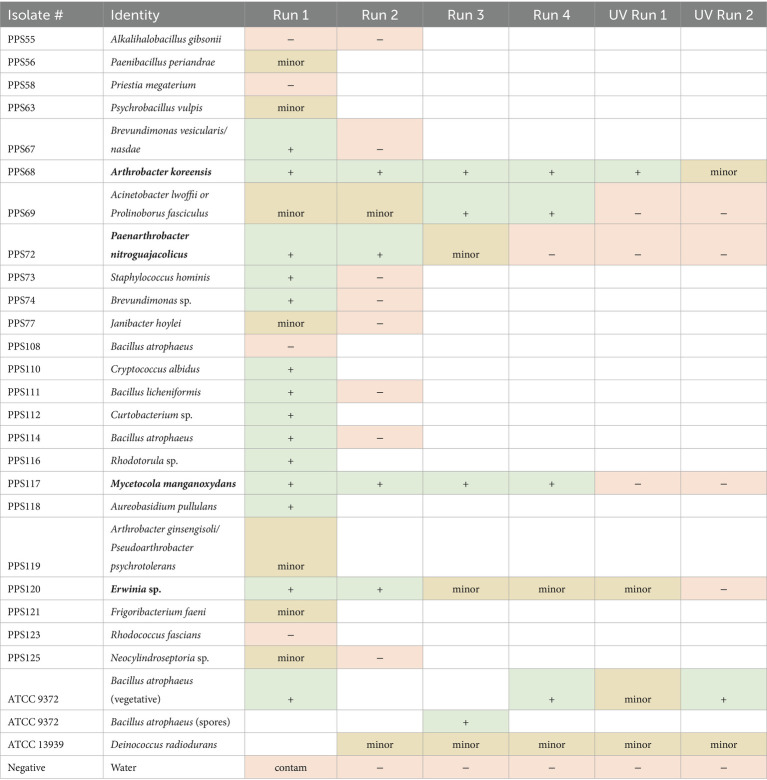

Growth is denoted by a plus sign and is shaded green. No detectable growth or obvious contamination based on morphology and color are denoted by a negative sign or “contam,” respectively, and are shaded red. Samples which demonstrated minor growth are denoted “minor” and are shaded yellow. Strains in bold were selected for whole genome sequencing.

After proton exposure, the resistant microbes were exposed to ultraviolet (UV_254_) radiation, representative of another type of space-relevant stressors. UV is not only relevant as a space environmental condition but has been considered as a sterilization procedure for “break-the-chain” PP workflows in the Mars Sample Return mission (Schuerger and Moores, 2023). In the UV experiment, the five most resistant microbes from the third run were exposed to 254 nm UV radiation at an intensity of 80 W/m^2^ for either UV Run 1 or 2 (5 or 10 min, respectively). While UV exposure was effective at killing most of the microbes tested, strain PPS120 (*Erwinia* sp.) exhibited minor survival. The minor growth was determined based on the results where one to three colonies were seen on one out of two samples exposed. In addition, survival of the strain PPS68 (*A. koreensis*) was recorded, when exposed to either 5 or 10 min of UV radiation. Positive controls (*B. atrophaeus* spores and *D. radiodurans*) tested exhibited minor growth.

Based on the resistance profiles observed, four strains were sequenced for WGS, including strains PPS68, PPS72, PPS117, and PPS120. Based on 16S rRNA gene sequencing these strains were identified as *Arthrobacter koreensis*, *Paenarthrobacter nitroguajacolicus*, *Mycetocola manganoxydans*, and *Erwinia* sp., respectively. However, WGS analysis identified the four isolates only to the genus level, except for strain PPS68. The WGS analysis suggests that three of the isolates may represent novel species, (Tables 3,4). Further taxonomic analysis was performed using ANI analysis.

**Table 3:** Genome features of assembled genomes of 4 selected microbes.

Genome features	*Mycetocola* sp. PPS117	*Erwinia* sp. PPS120	*Arthrobacter koreensis* PPS68	*Paenarthrobacter* sp. PPS72
Taxonomy ID	699879	558	199136	211146
Size (bp)	3,284,942	4,562,365	3,466,751	4,894,810
GC content (%)	63.9	54.5	65.9	62.1
N50 (bp)	366,358	122,723	204,164	93,542
L50 (bp)	4	14	6	17
Number of Contigs (with PEGs)	39	126	77	156
Number of Subsystems	247	337	277	283
Number of Coding Sequences	3,170	4,546	3,210	4,850
Number of tRNAs	48	57	47	47
Number of rRNAs	5	3	5	4

**Table 4:** ANI summarization.

Genome	Closest accession number	Closest organism	Strain	ANI %	Mapped fragments	Total fragments	Comparison genus
PPS68	ASM3579223v1	*Arthrobacter koreensis*	Gar NS 3	98.9836	1090	1134	*Arthrobacter*
PPS72	ASM4067646v1	*Paenarthrobacter nitroguajacolicus*	LAR2-1-1.1	88.6531	1188	1573	*Paenarthrobacter*
PPS72	ASM28106v1	*Arthrobacter* sp.	M2012083	88.2614	1148	1573	*Arthrobacter*
PPS117	ASM1463645v1	*Mycetocola zhadangensis*	CGMCC 1.12042	82.9025	698	1086	*Mycetocola*
PPS120	ASM3962185v1	*Erwinia* sp.	HDF1-3R	93.8774	1310	1475	*Erwinia*

### 3.1 Genome characteristics and relatedness in selected cleanroom strains

The 16S rRNA analysis identified the closest taxonomic affiliations of the query strains based on sequence similarity to known type strains. Strain PPS117 showed 99.03% similarity to *Mycetocola zhadangensis* ZD1-4. Strain PPS120 exhibited 98.56% similarity to *Erwinia tasmaniensis* Et/199. Strain PPS68 shared 99.47% similarity with *Arthrobacter luteolus* CF-25. Strain PPS72 exhibited 99.93% similarity to *Paenarthrobacter nitroguajacolicus* JCM 14115.

The GTDB-Tk ani_rep command was used to compare query genomes against a comprehensive reference dataset of 113,104 bacterial genomes, providing detailed assessments of genomic similarity through Average Nucleotide Identity (ANI) and alignment fractions. For strain PPS68, high ANI matches were observed and identified as *A. koreensis* (GCF_009193255.1) since ANI was 98.7% and 0.9317 alignment fraction. Strain PPS72 shows highest matches (88.65 and 88.26%) with *Paenarthrobacter nitroguajacolicus* (GCF_001375615.1) and *Arthrobacter* sp. (GCF_000281065.1). Strain PPS120 showed a closest match to *Erwinia* sp. (GCF_900068895.1) with an ANI of 94.1% and an alignment fraction of 0.8363, though it did not meet the threshold for a close match. For strain PPS117, ANI analysis found no significant matches, despite a 16S rRNA similarity of 99.0% with *M. zhadangensis* ZD1-4, indicating the potential for a novel species. However, according to FastANI, the closest match for the strain PPS117 was with *M. zhadangensis* (82.9%, GCF_014636455.1) (Table 4; Supplementary Table 3).

### 3.2 Comparative genomics of 4 selected cleanroom strains

**Figure 1:**
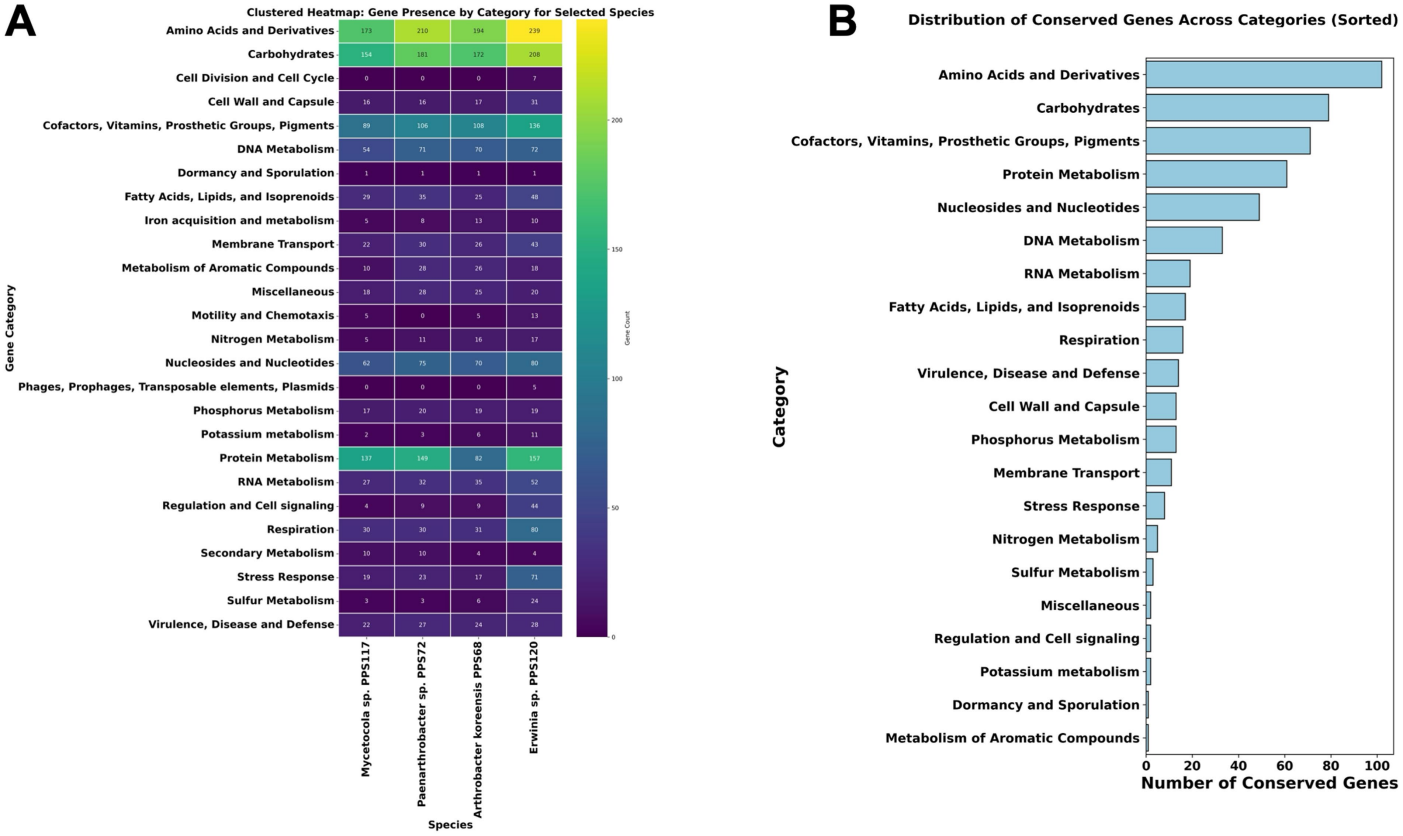
**(A)** Clustered heatmap of gene presence across functional categories in four bacterial species. This heatmap illustrates the distribution of gene counts across 25 functional categories for four bacterial species: *Arthrobacter koreensis PPS68*, *Erwinia* sp. *PPS120*, *Mycetocola manganoxydans PPS117*, and *Paenarthrobacter nitroguajacolicus PPS72*. Gene categories include pathways related to metabolism, stress response, dormancy, and more. The color gradient reflects gene abundance, with higher counts represented by lighter colors. This visualization highlights both shared and distinct genetic characteristics among the species, shedding light on their potential functional adaptations to various environmental conditions. **(B)** The bar chart represents the distribution of conserved genes across different functional categories identified in the core genome shared by the analyzed bacterial species. Categories such as Amino Acids and Derivatives, Carbohydrates, and Cofactors, Vitamins, Prosthetic Groups, and Pigments exhibit the highest conservation levels, indicating their essential roles in fundamental metabolic and cellular processes. The chart provides insights into the functional significance of conserved pathways and their contribution to bacterial survival and adaptation in varied environments. The heatmap uses a color gradient to indicate gene count, ranging from dark blue (low) to green (high) and yellow (highest).

The functional analysis identified 1,990 genes, observed across four bacterial species, which were classified into various function-based categories according to RAST (Figure 1A; Supplementary Table 4). In this dataset, 522 roles of genes were found to be conserved across four bacterial species, constituting ~26% of the total dataset. These conserved genes form part of the core genome shared by four species analyzed (Figure 1B; Supplementary Table 5). The conserved genes have functional annotations predictive of their role in regulating essential pathways like, carbohydrate metabolism, amino acid biosynthesis, protein processing, and cofactor and vitamin metabolism. Moreover, these genes are also related to the processes, nucleotide synthesis, DNA and RNA metabolism, and respiration which are consistently maintained, underscoring the fundamental requirements for cellular maintenance and energy generation. Additionally, several genes linked to membrane transport, lipid biosynthesis, and stress defense mechanisms were conserved, reflecting their important role in maintaining structural integrity and adaptive potential under dynamic environmental conditions.

### 3.3 Variation in pathway specialization in selected 4 cleanroom strains

Despite the conservation of core genes, variations in specific metabolic pathways were observed. Notably, carbohydrate metabolism genes showed significant diversity in these 4 organisms (Supplementary Table 4). *Erwinia* sp. PPS120 exhibited a broader set of genes associated with sugar utilization, indicating metabolic flexibility and the ability to exploit diverse carbon sources. Conversely, *A. koreensis* sp. PPS68 and *Paenarthrobacter* sp. PPS72 had more specialized genes in some pathways (Supplementary Table 4), which may reflect adaptations to specific environments with limited substrate diversity.

### 3.4 Stress response functions in selected 4 cleanroom strains

Around 90 genes were directly reported in the stress response category in RAST annotations, with an additional 463 genes identified as possible stress-response related. Out of these 553 functional entries, 21% were conserved across all four species and were mainly associated with stress response, membrane permeases, transporters, efflux pumps, resistance genes, antimicrobial genes, heat shock proteins and DNA repair genes.

### 3.5 Survival in harsh conditions

All species carry genes related to oxidative stress responses and detoxification, that might help them enhance survival in challenging environments. The presence of glutathione-dependent detoxification pathways in *Erwinia* sp. PPS120, for instance, may highlight its adaptability to oxidative environments. Compared to the other 3 cleanroom strains, *A. koreensis* PPS68 also displayed a robust set of stress response genes, including those for oxidative damage management and membrane stability, which may be contributing to its resilience to desiccation, radiation, and extreme temperatures. To cross-validate the genes that might be contributing to the radiation resistance in *A. koreensis* PPS68, we conducted further comparisons of these cleanroom strains with literature searched radioresistant and radiosensitive strains predominantly belonging to the phylum *Actinobacteria.*

### 3.6 Comparison of predicted resistome of *A. koreensis* PPS68 to radioresistant and radiosensitive strains

**Figure 2:**
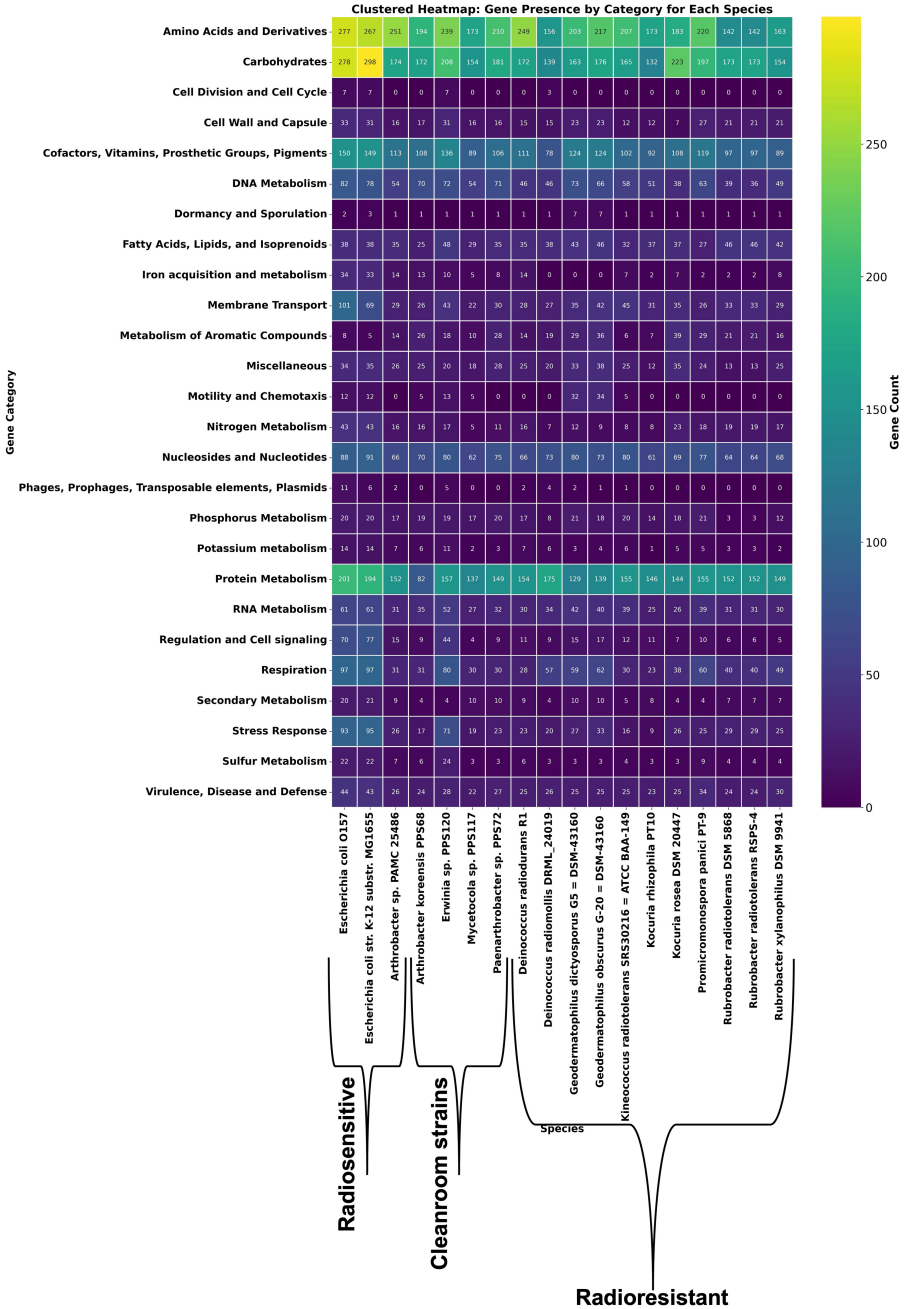
Clustered heat-map of gene counts in 26 functional categories across literature based radioresistant, radiosensitive representatives and clean-room isolates. Rows represent functional gene categories; columns represent individual bacterial genomes ordered as radiosensitive strains, clean-room isolates (including *Arthrobacter* sp. PPS68) and reference radioresistant taxa. Each cell contains the number of genes assigned to the corresponding category in the indicated genome. The heatmap uses a color gradient to indicate gene count, ranging from dark blue (low) to green (high) and yellow (highest).

**Figure 3:**
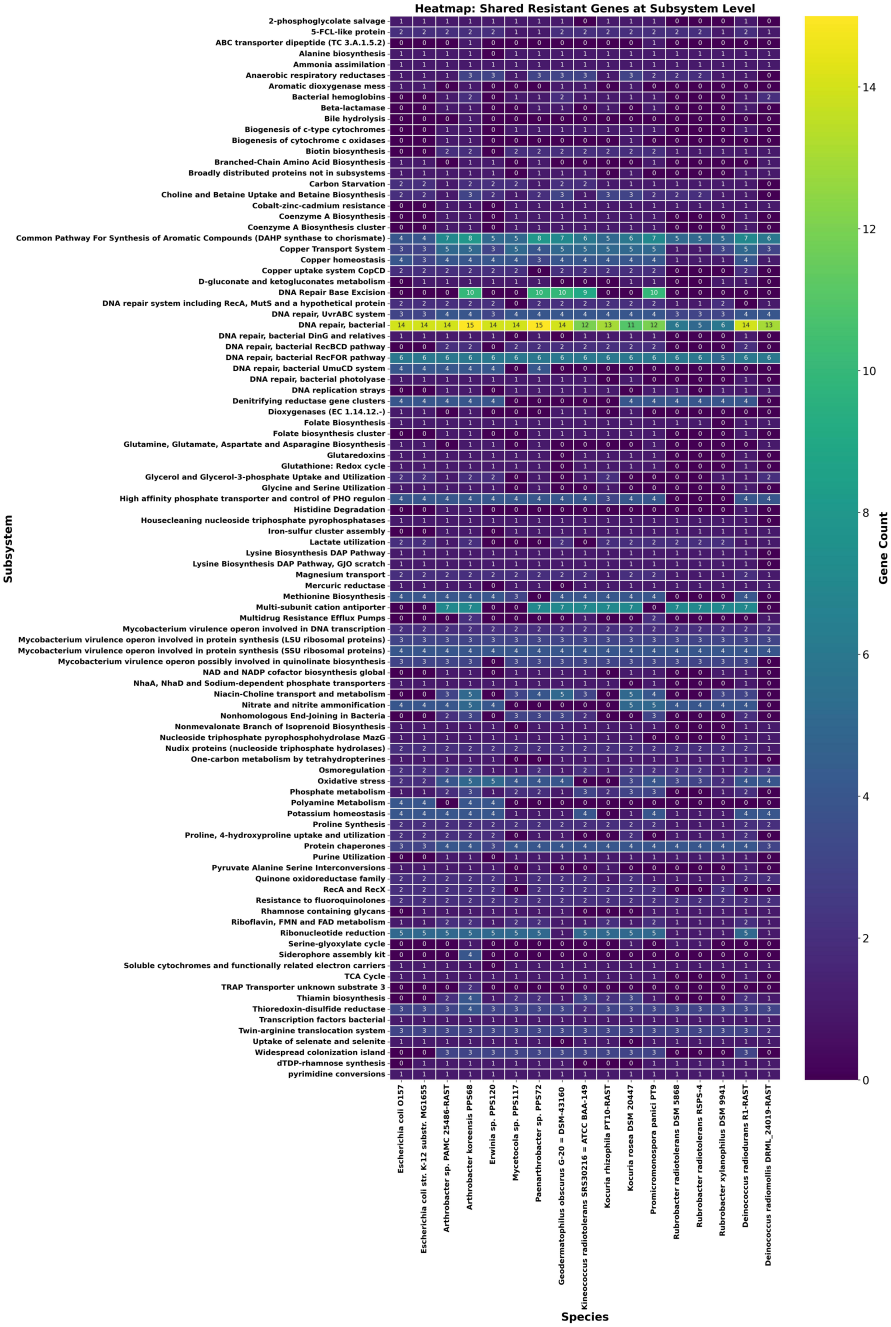
Heat-map of resistance-associated functions shared between *Arthrobacter* sp. PPS68 and at least one radioresistant comparator. Rows list individual resistance-related subsystems; columns list 11 radioresistant and 3 radiosensitive actinobacterial genomes together with *Arthrobacter* sp. PPS68 and other cleanroom strains. The heatmap used a color gradient to indicate gene count for each function, ranging from dark blue (low) to green (high) and yellow (highest).

Building on the above comparative overview of stress-response related functional annotations, a resistomics analysis was conducted by selecting literature searched 11 radioresistant strains of phylum *Actinobacteria* and 3 probable radiosensitive strains which includes a strain PAMC 25486 of *Arthrobacter* sp. (Figure 2; Supplementary Table 4). Further, by using Pan-ResistomeFinder, we have identified 220 PPS68 roles present in at least one additional radio-resistant strain (Figure 3). These functional roles include multiple oxidoreductases, membrane-associated efflux pumps, and DNA-repair enzymes, underscoring a conserved molecular machinery that may underpin the high radiation and desiccation tolerance shared across the resistant panel. Conversely, only 26 PPS68 roles/genes remained after all genes found in the sensitive controls were subtracted; this exclusive subset pinpoints candidate determinants that are retained by resistant strains yet absent from radiation-sensitive taxa, and therefore may contribute most directly to the extreme-tolerance phenotype observed in *A. koreensis* PPS68 (Supplementary Table 6). This analysis supports the prediction that PPS68 possesses an expanded DNA repair capacity, characterized by an augmented base-excision repair (BER) module operating in tandem with non-homologous end-joining (NHEJ) ligases (LigC/LigD) and accessory polymerases. Together, these components constitute a multi-layered strategy for resolving radiation-induced DNA damages. Concurrently, osmoprotection and redox balance in PPS68 appear to be regulated by dual glycine-betaine uptake and synthesis systems, thiamin-precursor importers, and folate-cycling enzymes Supplementary Table 7. In parallel, detoxification and adaptive signaling may be supported by multidrug efflux pumps, a c-di-GMP-modulating diguanylate cyclase/phosphodiesterase, and nitrite/phosphate regulatory elements (Supplementary Table 6).

## 4 Discussion

Bacterial and fungal isolates (*n* = 82) were recovered from built environments of various cleanliness levels and a subset was screened using increasingly harsh conditions mimicking some features of spaceflight and identified using sequencing of ribosomal RNA genes. A modular method was adapted from the literature to expose dried microbes to proton radiation in a clean and efficient manner to rapidly screen microbial isolates. The method used to expose microorganisms to proton radiation in this study was adapted from formerly published methods of exposing dried microbes to proton radiation in a relatively clean and efficient manner (Schuerger et al., 2003; Moeller et al., 2012; Cortesão et al., 2019; Hase et al., 2021). It can be modified to change many different features of experimental conditions, including what microbes are tested, the physical substrate the microbes are applied to, the density of the microbial population, the exposure/stressor employed, and the quantification method.

Using this platform, we have identified under-studied, non-sporulating isolates with resistance to ionizing radiation. Four isolates with the greatest tolerance for radiation were further characterized using WGS and comparative genomics analysis. Many of the genera detected in our study are consistent with taxa that have been detected in other NASA cleanrooms studies (Mahnert et al., 2015; Dworkin et al., 2017; Regberg et al., 2020; Hendrickson et al., 2021). Of note are microorganisms from the genera *Acinetobacter*, *Bacillus*, *Brevibacillus*, *Erwinia*, *Paenibacillus*, *Micrococcus*, *Staphylococcus*, *Pseudomonas*, *Cladosporium*, and *Penicillium*. Although prior studies have primarily analyzed cleanroom microbial communities using cultivation-independent molecular methods, the prevalence of these genera across multiple detection methods indicates that they are prevalent within cleanroom environments, irrespective of the location within the United States, are more easily sampled than other contaminants, or both.

Since microbes were isolated primarily from floors and surfaces of human-used, built environments, it is unsurprising that many microbes identified were associated with soil or human skin (Flowers and Grice, 2020; Philippot et al., 2023). Although many of these microbes have been poorly characterized, some of them have been previously identified as extremophiles. Specifically, species of the genus *Brevundimonas* and *Kocuria* have demonstrated the ability to survive in simulated Martian conditions (Dartnell et al., 2010; Vallalar, 2012). Furthermore, some genera, including *Deinococcus* and *Janibacter*, have been associated with resistance to certain stressors like ultraviolet radiation within the space environment (Shivaji et al., 2009; Ho et al., 2016). Finally, some of the genera identified, including those from the genera *Alkalihalobacillus*, *Bacillus*, *Brevibacillus*, *Calidifontibacillus*, *Fictibacillus*, *Lederbergia*, *Paenibacillus*, *Priestia*, *Psychrobacillus*, *Pseudogracilibacillus*, and *Streptomyces*, are known to form endospores which are capable of withstanding extreme environmental conditions and would likely be in the recoverable population from the NSA (Dietz and Mathews, 1971; Ash et al., 1993; Shida et al., 1996; Logan et al., 2009; Park et al., 2018; Adiguzel et al., 2020; Gupta et al., 2020; Rodríguez et al., 2020) Likely these documented, spore forming microbes did not demonstrate survival in our studies because they were exposed in a vegetative state. These conclusions further support previous findings that cleanrooms house many microbes that could be of potential risk to NASA missions as they relate to PP (Stieglmeier et al., 2009; Ghosh et al., 2010). GTDB-Tk ani_rep shows PPS68 at 98.7% ANI to *Arthrobacter koreensis* DSM 16760, the alkalitolerant soil type-species from Daejeon, Korea (Lee et al., 2003); its actual FastANI hit, strain Gar NS 3, was taken from bovine skin swabs in Guwahati, India and carries no irradiation data (NCBI BioSample: SAMN29049796). The strain PPS72 shares just 88.7% ANI with *Paenarthrobacter nitroguajacolicus* strain HG (GCF_001375615.1), a soil bacterium for which the exact source of isolation was not reported, but which is characterized by its ability to utilize papaverine an opium alkaloid antispasmodic drug as the sole carbon source (NCBI BioSample: SAMEA3310068). PPS120’s closest genome (94.1% ANI) is *Erwinia* sp. ErVv1 (GCF_900068895.1), a grape-vine (*Vitis vinifera*) endophyte from Italy (NCBI BioSample: SAMEA3216243). For PPS117, FastANI recovered only 82.9% identity to *Mycetocola zhadangensis* CGMCC 1.12042 (BMEK00000000.1), the type strain isolated from snow on the high-UV Zhadang Glacier, Tibetan Plateau, China (Shen et al., 2013), and no published radiation assays exist for this species.

The conserved genes in the cleanroom resistant strains are part of the core genome and can be involved in several functions like DNA repair and stress tolerance. For instance, DNA repair genes like RecA and the UvrABC excinuclease system provide protection against UV and oxidative DNA damage (Lenhart et al., 2012; Sinha et al., 2020; Kalogiannis and Eyre-Walker, 2024). Heat shock proteins, such as the 16 kDa heat shock protein A, stabilize and refold proteins during thermal stress, ensuring cellular functionality (Trilling et al., 2011; Srivastava et al., 2013). Furthermore, genes involved in oxidative stress and metal resistance, such as Alkyl hydroperoxide reductase and CopD, enhance resilience against reactive oxygen species and metal toxicity were present, facilitating adaptation to diverse environments (Hu and Zhao, 2007; Krishnamurthy et al., 2024; Ramnarine et al., 2024). *Erwinia* sp. PPS120 had an extensive array of stress response genes, particularly those involved in glutathione-dependent detoxification pathways, such as S-(hydroxymethyl)glutathione dehydrogenase and S-formylglutathione hydrolase (Chen et al., 2016; Osman et al., 2016). This predicts enhanced oxidative stress management, providing a survival advantage in environments prone to high oxidative damage. Additionally, all four species possessed the DedA protein for selenate and selenite transport, predictive of adaptations to environments with elevated selenium levels (Chen et al., 2016).

Specifically, *A. koreensis* PPS68 has reported notable resistance to both proton radiation and UV radiation. It is difficult to compare across studies where *Bacillus* spores have been exposed to protons (Moeller et al., 2012), but results from Run 3 preliminarily indicated that *A. koreensis* PPS68 was less resistant to radiation than *B. atrophaeus* spores (data not shown). While little is known about this strain of *A. koreensis*, previous literature suggests tolerance of both desiccation and alkaline conditions, and these features make it an organism of PP concern with the potential to survive the extreme conditions of space environments (Lee et al., 2003; Manzanera et al., 2015). *A. koreensis* PPS68 had several unique genes which were absent in the other cleanroom species genomically compared. Key genes include Thiol peroxidase-Tpx-type (EC 1.11.1.15), essential for detoxifying peroxides and protecting against oxidative damage induced by radiation (Lushchak, 2001). Multidrug resistance efflux pumps, such as the Acriflavin resistance protein and Multi antimicrobial extrusion protein, are known for exporting harmful compounds, enhancing oxidative stress management (Bogomolnaya et al., 2013). Thioredoxin-disulfide reductase maintains protein stability and supports recovery under stress (Kern et al., 2003). Additional genes like Sialidase (EC 3.2.1.18) may contribute to membrane stability (Kim et al., 2011), while the Histidine transport permease ensures nutrient acquisition under limited conditions (Zhang et al., 2015; Beetham et al., 2024). Stress response regulators with GGDEF and EAL domains modulate biofilm formation and stress responses, supporting survival in extreme environments (Rajeev et al., 2014). Siderophore biosynthesis and transport proteins (e.g., DesA, DesB, DesC, DesD) might also have roles in stress management because siderophores scavenge iron, which is crucial for many cellular processes, including those involved in stress responses. Siderophores play a critical role in mitigating radiation-induced oxidative damage by limiting free intracellular iron and preventing Fenton chemistry, a mechanism observed in extremophiles like *Deinococcus radiodurans* and potentially mirrored in cleanroom isolates (Daly, 2009; Krisko and Radman, 2013). In contrast, the presence of unique radiation and stress tolerance related genes does not confirm that they can be the contributing factor for their resistance until supported by further wetlab analysis. Thus, we tried to further validate these genomic predictions for their importance in radiation resistance through detailed comparative genomic analysis by including literature proven radiation resistant strains of phylum *Actinomycetota.*

Excluding genes shared with *E. coli* and a radio‑sensitive *Arthrobacter* strain revealed a distinct subset of genes in PPS68 that is present in at least one radiotolerant reference and absent from all three sensitive comparators (Supplementary Table 6). This genomic complement encodes a PhoU homolog that may regulate phosphate uptake during γ‑irradiation (diCenzo et al., 2017); the NHEJ ligase LigC, which may couple with LigD to re‑seal double‑strand breaks (Bhattarai et al., 2014); and a formate/nitrite exporter that mitigates nitrosative stress (Maeda et al., 2015). Osmoprotection and redox balance might be reinforced by dual glycine‑betaine uptake/synthesis genes (OpuD and betaine aldehyde dehydrogenase) (Boch et al., 1996; Kappes et al., 1996), whereas a Na^+^‑coupled MATE antiporter and an Acriflavin protein can remove toxic metabolites (Omote et al., 2006; Fischer and Kandt, 2013). An expanded base‑excision repair arsenal including polymerase‑like MT3142, Ku, LigD, LigC, polymerase I, and multiple glycosylases and endonucleases might be capable to facilitate rapid repair of clustered DNA breaks, while quinate/shikimate‑5‑dehydrogenase supports antioxidant and folate biosynthesis (Krokan and Bjørås, 2013; Marienhagen, 2025). Stress signalling in the strain may be modulated by a PAS‑domain GGDEF/EAL diguanylate cyclase/phosphodiesterase (Rajeev et al., 2014) whereas Vanillate O-demethylase oxidoreductase may dissipate reactive species and regenerate NAD^+^; and the ABC dipeptide transporter DppD could be responsible for acquisition of amino acids and peptides needed for DNA repair‑protein synthesis (Xu et al., 2021; Hu et al., 2024) in the presence of radiation stress conditions. Collectively, these functions absent from all sensitive genomes constitute an integrated network of DNA repair, osmotic and redox homeostasis, detoxification, and adaptive signalling that underpins their probable role in PPS68’s exceptional radiotolerance than rest of the strains. It is interesting to note that *Paenarthrobacter* sp. PPS72 also has 17 of these genes common whereas other two cleanroom strains share 2 for each.

Although this study has focused on a single type of radiation exposure (protons), there are other stressors that space-faring microbes will be exposed to, including: temperature extremes, broad spectrum ultraviolet (UV) radiation, microgravity, chemical assault, and starvation. Much of the benefit of this study was developing protocols and setting baseline exposures to better prepare for more stringent future studies. In addition, the UV experiment we performed was simple and used a common lab UV crosslinker which has a much higher intensity, perhaps as much as two orders of magnitude higher, than the intensity of 254 nm wavelength light in space. Therefore, future work from this proposal would include tightening the space-relevance of experimental parameters and performing stringent individual and combination studies to understand the ability of these microbes to survive the effects of multiple stressors (Smith et al., 2009; Schuerger, 2024).

The results of this study emphasize the necessity to modernize PP policies by shifting from spore burden analysis to a broader evaluation of microbes, including non-spore-formers, to address their ability to survive and adapt to space-related stressors. Molecular methods enhance the understanding of microbial diversity, especially non-culturable organisms. However, characterizing cultivable non-spore-forming microorganisms is essential for refining decontamination strategies. Investigations into the effects of desiccation, vacuum, and proton radiation on non-sporulating cleanroom isolates highlight critical gaps in knowledge regarding microbial survival, adaptation, and resilience in oligotrophic environments like spacecraft assembly facilities and cleanrooms. This information is vital for mitigating microbial risks in robotic and crewed missions beyond low Earth orbit, where extreme conditions may impact microbial behavior, crew health, and life support systems. Furthermore, genomic and functional analyses of high-risk strains provide insights to develop targeted mitigation strategies. These findings have broader implications for astrobiology, space biology, and environmental monitoring, ensuring PP, mission success, and the preservation of scientific objectives.

This study highlights the necessity of modernizing PP policies to include non-spore-forming microbes in bioburden assessments, offering broader applications beyond spacecraft. Insights into microbial survival and adaptation to extreme conditions are relevant to contamination control in pharmaceutical, medical, and semiconductor industries, enhancing sterility, product integrity, and operational safety.

## Data Availability

The sequencing data generated in this study have been deposited in the NCBI Sequence Read Archive (SRA) under BioProject accession PRJNA1211674. The raw sequencing reads for the four microbial genomes are available under the following SRA accessions: Strain PPS72: SRX27599488; Strain PPS68: SRX27599487; Strain PPS120: SRX27599486, Strain PPS117: SRX27599485.
