# Supplementary material for: Effects of simulated space environmental conditions on cleanroom microbes
Source: Front Microbiol. 2025 Aug 19;16:1600106. doi: 10.3389/fmicb.2025.1600106 (PMC12404038; doi:10.3389/fmicb.2025.1600106)
Supplement: Supplementary file 1 [file Data_Sheet_1.zip › Supplementary Material/Supplemental Methods.pdf]

PCR was conducted using an Applied Biosystems SimpliAmp Thermalcycler (A41192; Thermo Fisher Scientific, Waltham, MA). Primers were resuspended using the Dilution Calculator on the IDT website. For bacterial 16S rRNA gene amplification, the mixture included 1 µL of 10 mM Deoxynucleotide (dNTP) Solution Mix (N0447S; New England Biolabs, Ipswich, MA), 1 µL of 20 µM 16S rRNA For primer (IDT ReadyMade primer 51-01-19-06), 1 µL of 20 µM 16S rRNA Rev primer (IDT ReadyMade primer 51-01-19-07), template DNA to a concentration of approximately 100 ng, 10 µL of 5X One*Taq* Standard Reaction Buffer (New England Biolabs, Ipswich, MA), 0.2 µL of One*Taq* DNA polymerase (M0480L; New England Biolabs, Ipswich, MA), and sterile water up to 50 µL final volume. The settings used were as suggested by NEB and IDT and are as follows: an Initial Denaturation at 94 °C for 30 seconds; 35 cycles including Denaturation at 94 °C for 30 seconds, Annealing at 52 °C for 1 minute, and Elongation at 68 °C for 1 minute; and a Final Extension at 68 °C for 5 minutes.

For amplification of the fungal ITS region, the mixture included 1 µL of 10 mM dNTP Solution Mix (N0447S; New England Biolabs, Ipswich, MA), 1 µL of 20 µM ITS5 forward primer (IDT, Coralville, IA), 1 µL of 20 µM ITS4 reverse primer (IDT, Coralville, IA), template DNA to a concentration of approximately 100 ng, 10 µL of 5X One*Taq* Standard Reaction Buffer (New England Biolabs, Ipswich, MA), and 0.2 µL of One*Taq* DNA polymerase (M0480L; New England Biolabs, Ipswich, MA), and sterile water up to 50 µL final volume. The settings used were as described in (Schoch *et al.*, 2012) and are as follows: an Initial Denaturation at 95 °C for 10 minutes; 35 cycles including Denaturation at 95 °C for 15 seconds, Annealing at 52 °C for 30 seconds, and Elongation at 72 °C for 1.5 minutes; and a Final Extension at 72 °C for 7 minutes.

PCR amplification products were visualized using an Invitrogen E-Gel Powersnap System (G8352ST; Waltham, MA). In short, 2% agarose E-Gels with SYBR Safe (Invitrogen, Waltham, MA) were loaded into the system. 5 µL of E-Gel 1 KB Plus DNA Ladder (Invitrogen) was loaded into the corresponding well. Then 2 µL of E-Gel Sample Loading Buffer (Invitrogen) was mixed with 5 µL of PCR product and loaded into designated wells. The gel was run under the pre-set condition for 0.8–2% E-Gels for 26 minutes. Gels were then visualized with UV light and images were captured of each. The 16S rRNA amplification product was approximately 1500 bp, while the ITS amplification product was approximately 500 bp. Samples that failed to show bands at expected sizes underwent a repeated PCR attempt. Only samples with clear bands of expected size were purified in the following step.

DNA purification was conducted with the Zymo Research DNA Clean & Concentrator -100 Kit (Cat No. D4030; Irvine, CA). Zymo-Spin collection reservoirs were discarded and the remaining column was placed into a labeled collection tube. For 50 µl PCR samples, the PCR product was mixed with 250 µL of DNA Binding Buffer and gently pipetted up and down to mix. Material was transferred into a column and centrifuged at maximum speed for 1 minute. A volume of 600 µL of DNA Wash Buffer was added to the columns and centrifuged at maximum speed for 1 minute. Centrifugation was repeated to clear the column of residual fluid. The columns were placed into new Eppendorf tubes and then 50 µL of water was added directly onto the column. Samples were allowed to sit for 1 minute to ensure DNA on the column dissolved into the water. Tubes were then centrifuged at maximum speed for 1 minute before the column was discarded. Finally, DNA concentration was measured using a Thermo Scientific Nanodrop Lite (Waltham, MA).
