# Supplementary material for: Effects of simulated space environmental conditions on cleanroom microbes
Source: Front Microbiol. 2025 Aug 19;16:1600106. doi: 10.3389/fmicb.2025.1600106 (PMC12404038; doi:10.3389/fmicb.2025.1600106)
Supplement: Supplementary file 1 [file Data_Sheet_1.zip › Supplementary Material/Supplementary Table 1.pdf]

Supplementary Table 1: Identification of Cleanroom-Associated Microbes

Identifier	Location	Sample	Substrate	Identification
PPS47	E	Air		*Staphylococcus* sp.
PPS48	E	Air		*Neomicrococcus lactis*
PPS49	E	Air		*Fictibacillus* sp.
PPS50	E	Air		*Metabacillus idriensis*
PPS51	E	Air		*Knoellia flava*
PPS52	E	Air		*Brevibacillus borstelensis*
PPS53	E	Air		*Deinococcus ficus*
PPS54	E	Air		*Knoellia flava*
PPS55	A	Floor	wet swab	*Alkalihalobacillus gibsonii*
PPS56	A	Floor	wet swab	*Paenibacillus periandrae*
PPS57	E	Table	wet swab	**No significant similarity found**
PPS58	B	Table	wet swab	*Priestia megaterium*
PPS59	B	Table	wet swab	*Lederbergia* sp.
PPS60	B	Table	wet swab	*Pseudarthrobacter* sp. **(high background)**
PPS61	B	Table	wet swab	**No ID >97%**
PPS62	B	Table	wet swab	*Bacillus* sp.
PPS63	B	Table	wet swab	*Psychrobacillus vulpis*
PPS64	B	Table	wet swab	**No ID >97% (poor quality)**
PPS65	B	Table	wet swab	*Streptomyces* sp.
PPS66	B	Floor	wet swab	*Micrococcus* sp.
PPS67	B	Floor	wet swab	*Brevundimonas vesicularis/nasdae*
PPS68	B	Floor	wet swab	*Arthrobacter koreensis*
PPS69	B	Floor	wet swab	*Acinetobacter lwoffii or Prolinoborus fasciculus*
PPS70	B	Floor	wet swab	*Microbacterium pumilum* **(non-specific)**
PPS71	B	Floor	wet swab	*Pseudomonas glycinis/koreensis* **(non-specific)**
PPS72	B	Floor	wet swab	*Paenarthrobacter nitroguajacolicus*
PPS73	B	Floor	wet swab	*Staphylococcus hominis*
PPS74	B	Floor	wet swab	*Brevundimonas* sp.
PPS75	B	Floor	wet swab	*Pseudogracilibacillus endophyticus*
PPS76	B	Floor	wet swab	*Acinetobacter lwoffii or Prolinoborus fasciculus*
PPS77	B	Floor	wet swab	*Janibacter hoylei*
PPS78	C	Floor	wet swab	*Bacillus* sp.
PPS79	C	Floor	wet swab	*Bacillus* sp.
PPS80	C	Floor	wet swab	*Stenotrophomonas nematodicola* **(High background)**
PPS81	C	Floor	wet swab	*Acinetobacter lwoffii or Prolinoborus fasciculus*
PPS82	E	Table	dry swab	*Bacillus atrophaeus*
PPS83	E	Table	dry swab	*Bacillus atrophaeus/vallismortis*

PPS84	E	Table	dry swab	*Staphylococcus hominis*
PPS85	E	Table	dry swab	*Paenibacillus* sp.
PPS86	E	Table	dry swab	*Staphylococcus capitis/caprae*
PPS87	E	Table	dry swab	*Staphylococcus epidermidis*
PPS88	E	Table	wet swab	*Staphylococcus* sp.
PPS89	E	Table	wet swab	*Staphylococcus auricularis*
PPS90	E	Table	wet swab	*Bacillus* sp.
PPS91	E	Table	wet swab	*Staphylococcus* sp.
PPS92	E	Table	wet swab	*Bacillus* sp.
PPS99	E	Table	wet wipe	*Okibacterium* sp. **(poor quality)**
PPS100	E	Table	wet wipe	*Bacillus* sp.
PPS101	E	Table	wet wipe	*Bacillus atrophaeus*
PPS102	E	Table	wet wipe	*Curtobacterium allii*
PPS103	E	Table	wet wipe	**No significant similarity found (high background)**
PPS104	E	Table	wet wipe	*Bacillus atrophaeus*
PPS105	E	Table	wet wipe	*Pseudomonas* sp.
PPS106	E	Table	wet wipe	*Rhodotorula mucilaginosa*
PPS107	C	Table	wet wipe	*Bacillus atrophaeus*
PPS108	C	Table	wet wipe	*Bacillus atrophaeus*
PPS109	C	Table	wet wipe	**No significant similarity found (non-specific)**
PPS110	A	Table	wet wipe	*Cryptococcus albidus*
PPS111	A	Table	wet wipe	*Bacillus licheniformis*
PPS112	A	Table	wet wipe	*Curtobacterium* sp.
PPS113	A	Table	wet wipe	**No significant similarity found (non-specific)**
PPS114	B	Table	wet wipe	*Bacillus atrophaeus*
PPS115	B	Table	wet wipe	**No significant similarity found (high background)**
PPS116	B	Table	wet wipe	Yeast (*Rhodotorula* sp. **from microscopy**)
PPS117	B	Table	wet wipe	*Mycetocola manganoxydans*
PPS118	B	Table	wet wipe	*Aureobasidium pullulans*
PPS119	B	Table	wet wipe	*Arthrobacter ginsengisoli/Pseudoarthrobacter psychrotolerans*
PPS120	B	Table	wet wipe	*Erwinia* sp.
PPS121	B	Table	wet wipe	*Frigoribacterium faeni*
PPS122	B	Table	wet wipe	*Cladosporium* sp.
PPS123	B	Table	wet wipe	*Rhodococcus fascians*
PPS124	B	Table	wet wipe	*Penicillium crustosum*
PPS125	B	Table	wet wipe	Yeast (*Neocylindroseptoria* sp. **from microscopy**)
PPS126	B	Table	wet wipe	*Arthrobacter oryzae* **(high background)**
PPS 221	D	Floor	wet wipe	*Sphingomonas desiccabilis*

PPS222	D	Floor	wet wipe	*Cryptococcus albidus*
PPS223	D	Floor	wet swab	*Bacillus toyonensis/thuringiensis*
PPS224	D	Floor	wet swab	*Bacillus* sp.
PPS225	D	Floor	wet swab	*Brevibacillus* sp.
PPS226	D	Floor	wet swab	*Bacillus acidiceler*
PPS229	D	Floor	wet swab	*Bacillus acidiceler*
PPS230	D	Floor	wet swab	*Bacillus* sp.
