# Supplementary material for: Effects of simulated space environmental conditions on cleanroom microbes
Source: Front Microbiol. 2025 Aug 19;16:1600106. doi: 10.3389/fmicb.2025.1600106 (PMC12404038; doi:10.3389/fmicb.2025.1600106)
Supplement: Supplementary file 1 [file Data_Sheet_1.zip › Supplementary Material/Supplementary Table 2.pdf]

Supplementary Table 2: Experimental Details for Exposure Studies

Experiment	Description	Run 1	Run 2	Run 3	Run 4	UV Run 1	UV Run 2
**Kapton Coupon Preparation**	1-in² Kapton-HN film coupons sterilized using a 3870ELP Heidolph Tuttnauer benchtop sterilizer.	x	x	x	x	x	x
**Facility**	Microbiological work performed in a BSL-2 facility.	x	x	x	x	x	x
**Drying Process**	Samples dried for up to three days before radiation exposure.	x	x	x	x	x	x
**Control Types**	- **Media controls:** Exposed without bacteria.	x	x	x	x	x	x
	- **Ambient negative controls:** Not exposed to vacuum or radiation.		x	x	x	x	x
	- **Vacuum controls:** Subjected to vacuum (~1E-6 Torr) without irradiation.				x		
**Proton Radiation**	Conducted at Combined Environmental Effects Facility (CEEF).	x	x	x	x		
	Samples exposed to 100 keV protons under high vacuum (~1E-6 Torr).	x	x	x	x		
**Proton Fluences**	$2x10^{15}$ to $4x10^{15}$ p+/cm².	x	x	x	x		
**Sample Volumes**	- **First run:** 250 µL (no controls).	x					
	- **Subsequent runs:** 200 µL (included ambient unexposed controls).		x	x	x		
**Exposure Time**	10.7 to 36.5 hours depending on the run.	x	x	x	x		
**Vacuum Duration**	46 to 145 hours depending on the run.	x	x	x	x		
**UV Radiation**	Samples exposed to 254 nm light at 80 W/m² for 5 or 10 minutes using Spectroline XL-1500 UV Crosslinker.					x	x
**Processing**	UV-irradiated samples processed on the same day.					x	x
**Challenges**	Contamination, prolonged drying, and mid-run interruptions affected experimental outcomes.	x					
