# Supplementary material for: Effects of simulated space environmental conditions on cleanroom microbes
Source: Front Microbiol. 2025 Aug 19;16:1600106. doi: 10.3389/fmicb.2025.1600106 (PMC12404038; doi:10.3389/fmicb.2025.1600106)
Supplement: Supplementary file 1 [file Data_Sheet_1.zip › Supplementary Material/Supplementary Table 3.pdf]

Supplementary table 3. ANI results as tested by GTDB-Tk and FastANI tools

Strain #	Comparison Genus for FastANI	Closest Organism as per FastANI	GenBank accession # of closest species as per FastANI	Fast ANI %	FastANI Mapped fragments	FastANI Total fragments	Closest Organism as per GTDBTk	GenBank accession # of closest species as per GTDBTk	GTDBTk-ANI %	GTDBTk-Alignment Fraction
PPS68	*Arthrobacter*	*Arthrobacter koreensis*	GCF_035792235.1	98.9836	1090	1134	*Arthrobacter koreensis*	GCF_009193255.1	98.7	0.9317
PPS72	*Arthrobacter*	*Arthrobacter sp.* M2012083	GCF_000281065.1	88.2614	1148	1573	*Arthrobacter* sp	GCF_000281065.1	88.24	0.6293
PPS72	*Paenarthrobacter*	*Paenarthrobacter nitroguajacolicus*	GCF_001375615.1	88.6531	1191	1573	Not tested separately	-	-	-
PPS117	*Mycetocola*	*Mycetocola zhadangensis*	GCF_014636455.1	82.9025	698	1086	NR	-	-	-
PPS120	*Erwinia*	*Erwinia sp.* ErVv1	GCF_900068895.1	93.9318	1311	1475	*Erwinia* sp.	GCF_900068895.1	94.11	0.8363

- represents no data available, NR- no significant result
